# Supplementary material for: Disclosure of study funding and author conflicts of interest in press releases and the news: a retrospective content analysis with two cohorts
Source: BMJ Open. 2021 Jan 8;11(1):e041385. doi: 10.1136/bmjopen-2020-041385 (PMC7798706; doi:10.1136/bmjopen-2020-041385)
Supplement: Author's manuscript [file bmjopen-2020-041385.draft_revisions.pdf]

BMJ Open is committed to open peer review. As part of this commitment we make the peer review history of every article we publish publicly available.

When an article is published we post the peer reviewers' comments and the authors' responses online. We also post the versions of the paper that were used during peer review. These are the versions that the peer review comments apply to.

The versions of the paper that follow are the versions that were submitted during the peer review process. They are not the versions of record or the final published versions. They should not be cited or distributed as the published version of this manuscript.

BMJ Open is an open access journal and the full, final, typeset and author-corrected version of record of the manuscript is available on our site with no access controls, subscription charges or pay-per-view fees (http://bmjopen.bmj.com).

If you have any questions on BMJ Open's open peer review process please email info.bmjopen@bmj.com

BMJ Open

Disclosure of funding and conflicts of interest in press releases and the news: A retrospective observational study

Journal:	*BMJ Open*
Manuscript ID	bmjopen-2020-041385
Article Type:	Original research
Date Submitted by the Author:	06-Jun-2020

Complete List of Authors:	Sumner, Petroc; Cardiff University School of Psychology,<br>Schwartz, Lisa; Dartmouth Institute for Health Policy and Clinical Practice<br>Woloshin, Steven; VA Medical Center / Dartmouth medical school, medicine<br>Bratton, Luke; Cardiff University School of Psychology<br>Chambers , Christopher; Cardiff University School of Psychology

Keywords:	MEDICAL JOURNALISM, PUBLIC HEALTH, STATISTICS & RESEARCH METHODS

SCHOLARONE™
Manuscripts

I, the Submitting Author has the right to grant and does grant on behalf of all authors of the Work (as defined in the below author licence), an exclusive licence and/or a non-exclusive licence for contributions from authors who are: i) UK Crown employees; ii) where BMJ has agreed a CC-BY licence shall apply, and/or iii) in accordance with the terms applicable for US Federal Government officers or employees acting as part of their official duties; on a worldwide, perpetual, irrevocable, royalty-free basis to BMJ Publishing Group Ltd ("BMJ") its licensees and where the relevant Journal is co-owned by BMJ to the co-owners of the Journal, to publish the Work in this journal and any other BMJ products and to exploit all rights, as set out in our licence.

The Submitting Author accepts and understands that any supply made under these terms is made by BMJ to the Submitting Author unless you are acting as an employee on behalf of your employer or a postgraduate student of an affiliated institution which is paying any applicable article publishing charge ("APC") for Open Access articles. Where the Submitting Author wishes to make the Work available on an Open Access basis (and intends to pay the relevant APC), the terms of reuse of such Open Access shall be governed by a Creative Commons licence – details of these licences and which Creative Commons licence will apply to this Work are set out in our licence referred to above.

Other than as permitted in any relevant BMJ Author's Self Archiving Policies, I confirm this Work has not been accepted for publication elsewhere, is not being considered for publication elsewhere and does not duplicate material already published. I confirm all authors consent to publication of this Work and authorise the granting of this licence.

For peer review only

Disclosure of funding and conflicts of interest in press releases and the news: A retrospective observational study

Petroc Sumner, Professor, PhD[1]

Lisa M. Schwartz, MD, MS[2]

Steven Woloshin, MD, MS[2,3]

Luke Bratton, PhD[1]

Christopher D. Chambers, Professor, PhD[1]

[1]School of Psychology, Cardiff University, UK; sumnerp@cardiff.ac.uk

[2]The Dartmouth Institute for Health Policy and Clinical Practice, Lebanon, NH, USA

[3] The Lisa Schwartz Foundation for Truth in Medicine, Norwich, VT, USA

Corresponding author: Petroc Sumner, sumnerp@cardiff.ac.uk

Acknowledgements: This article is dedicated to the memory of Lisa Schwartz (1963-2018). Lisa inspired this investigation and produced the first version of the manuscript. Lisa died in November 2018. We believe this paper stays true to her spirit and intentions. We thank the following for data collection and related projects that informed our approach and interpretation here: Rachel Adams, Caitlin Argument, Amy Barrington, Jacky Boivin, Lewis Bott, Laura Benjamin, Hannah Coulson, Eleanor Corney, Bethan Dalton, Aimee Davies, Cecily Donnelly, Cameron Dunlop, Rebecca Emerson, Rose Fisher, Oliver Gray, Bethan Hughes, Katie John, Laura Jones, Sarah Mann, Olivia Manship, Hannah Maynard, Hannah McCarthy, Jack Ogden, Amy Parfitt, Naomi Scott, Lauren Stead, Christos Venetis, Solveiga Vivian-Griffiths, Eliza Walwyn-Jones, Claire Weeks, Leanne Whelan, Andrew Williams, Joe Wilton.

Funding - This project was supported by ESRC grant ES/M000664/1 and Wellcome grant 104943/Z/14/Z. The funders had no role in the analysis or writing of this paper.

Competing Interest Statement - The authors have no conflicts to declare.

Authorship All authors contributed to the planning, conduct and reporting of the work described in the article.

Data sharing Data are publicly archived at https://osf.io/apc6d/

Word count: 2400

For peer review only

Objectives. To examine how often study funding and author conflicts of interest are stated in in science and health press releases and in corresponding news; and whether disclosure in news is associated with disclosure in press releases. Second, to specifically examine disclosure rates in industry-funded studies.

Design. Retrospective quantitative content analysis.

Setting. Press releases about health, psychology or neuroscience research from research universities and journals from 2011 (n=996) and 2015 (n=254) and their associated news stories (n=1250 and 578).

Primary outcome measure. Mention of study funding and author conflicts of interest.

Results. In our 2011 cohort, funding was reported in 94% (934/996) of journal articles, 29% (284/996) of press releases and 9% (112/1250) of news. The corresponding figures for 2015 were similar: 84% (214/254), 52% (131/254) and 10% (58/578). A similar pattern was seen for the industry funding subset. If the press release reported study funding, news was more likely to: 22% if in the press release vs 7% if not in the press release (2011), RR 3.1 (95% CI: 2.1 to 4.3); for 2015, corresponding figures were 16% vs 2%, RR 6.8 (95% CI: 2.2 to 17). In journal articles, 27% and 22% reported a conflict of interest, while less than 2% of press releases or news ever mentioned these.

Conclusions. Press releases and associated news did not frequently state funding sources or conflicts of interest. Funding information in press releases was associated with such information in news. Given converging evidence that news draws on press release content, including statements of funding and conflicts of interest in press releases may lead to increased reporting in news.

Article Summary: Strengths and limitations of this study.

- How often press releases and news report study funding and conflicts of interest is assessed using a large cohort of press releases (1250) and news (1828) across two separate years.
- The association between news and press release reporting is also assessed.
- The study is correlational and retrospective

For peer review only - http://bmjopen.bmj.com/site/about/guidelines.xhtml

Introduction

Medical journals, funders, and academic institutions routinely call on researchers to disclose funding sources and financial conflicts of interest. Doing so promotes public trust in the research process and allows readers to decide whether industry entanglements merit heightened skepticism when interpreting results.

There are no corresponding disclosure requirements for research reported in the lay press: only 3% of the largest circulation US newspapers had an explicit policy about reporting industry funding of medical research [1]. Published reports have documented substantial underreporting of author conflicts of interest and industry funding in the lay media [2-4]. Such underreporting matters since many people - including physicians [5] - learn about the results of medical research from the news [6].

The majority of news stories about news health-related discoveries are stimulated by press releases from universities or academic journals. Several studies suggest that press releases may strongly influence the content of subsequent media coverage. For example, news stories were more likely to report absolute risks, intervention harms and study limitations when they were reported in the medical journal press release [3]. Similarly, causal claims from correlational data, exaggerated inference to humans from animal research and 'spin' in news stories - or caveats to mitigate such exaggeration - tracked with their presence or absence in corresponding press releases [7-13].

We analysed how often funding and conflicts of interest are mentioned in biomedical and health news stories and their corresponding journal and institution press releases. We examined whether the presence of such statements in news is associated with

For peer review only

the presence in press releases. We then specifically examine the subset of studies that had industry funding.

Methods

We analyzed two collections of health-related news stories, press releases and associated journal articles. This first contains 1250 news stories, 996 press releases and 996 associated journal articles [8,9]. This database was collated by selecting all the press releases related to human health published throughout 2011 from 8 leading international biomedical journals (Lancet, British Medical Journal (BMJ), Science, Nature, Nature Neuroscience, Nature Immunology, Nature Medicine and Nature Genetics) and 20 leading UK universities (The Russell Group). The corresponding journal article for each press release was sourced, as were subsequent news stories in mainstream print and internet outlets [8,9]. The second database [12,13] contains 1250 news stories, 254 press releases and 254 associated journal articles. This was collated by selecting press releases related to human health published between January and June 2015, from 26 universities (including the Russell Group) and 26 journals (10 in the BMJ group, 16 in the BMC group).

Press releases were identified from publicly available repositories (web pages or EurekAlert) or nonpublic sites for journalists (Nature Publishing Group provided us with free access to all their press releases). The inclusion criteria were: health-related topic (broadly defined to include all biomedical sciences, diet, lifestyle, psychology) based on a peer-reviewed published journal article (that we could access). In the 2011 set all eligible press releases were included. In the 2015 set, the contribution from each institution had been capped to 10 press releases, selected randomly if more than 10 were available (for feasibility reasons [12,13]).

For peer review only

For peer review only - http://bmjopen.bmj.com/site/about/guidelines.xhtml

To identify any print and online news stories related to each press release, we searched Lexis-Nexis, BBC.co.uk, uk.reuters.com, and Google with keywords up to 28 days after publication of the press release, and up to one week before (to allow for potential news before the embargo was lifted).

Funding - general

Journal articles, press releases and related news coverage were coded by research assistants using a pre-specified protocol, to extract information about study funding and the authors' reported conflicts of interest. Study funding was coded as *industry* (e.g. GlaxoSmithKline, Pfizer etc.), *government* (e.g. the research councils such as the NIH and US National Cancer Institute, MRC, BBSRC, ESRC etc.), *charity* (e.g. Wellcome Trust, Cancer Research UK, the British Heart Foundation etc.), *internal/other* (e.g. self- or university-funded), or *none mentioned*. The largest or first-mentioned source was always coded, and then industry funding if it was listed (see below).

Industry funding.

For the specific focus on industry funding, a study was designated industry-funded if any of the funding sources mentioned included industry, regardless of position in the list of funders.

Conflicts of interest

Coders looked for the "Conflict of interest" or "Competing interests" sections of the article and determined whether there was no declaration found, a declaration of no conflict of

For peer review only

interest (e.g. "The authors declare no conflict of interest”) or whether any author declared conflicts of interest (e.g. “Author X is a paid consultant to Y company).

The press releases and news stories were similarly coded for whether funding or conflicts of interest were reported, and whether the press release and news specifically mentioned industry funding. Each Press release and news was also coded for whether it mentioned conflicts of interest declared by the authors, mentioned that no conflicts of interest were declared or had no mention.

Coding reliability

For the 2011 set, a second research assistant independently coded a randomly selected sample of 28% of press releases and associated news (23% of total news stories). Observed agreement was 94% for coding the type of funding source, 92% for whether PR reported funding, 94% for whether news reported funding, 98% for the study’s conflict of interest statement, and 99% for both whether press release and news mentioned conflict of interest (we do not calculate kappa as it is unreliable when agreement is this high). For the 2015 set, a second research assistant independently coded all texts, and any discrepancies were subsequently highlighted and discussed to reach a consensus conclusion. A third research assistant arbitrated if disagreements remained (very rare).

Analysis

We first report analyses of all journal articles and associated press releases (n=996 for 2011, n=254 for 2015) descriptively, followed by descriptive analysis of news (n=1,250 news and 578 news, respectively). These are separated by year to illustrate the natural

For peer review only - http://bmjopen.bmj.com/site/about/guidelines.xhtml

range of fluctuation, rather than to examine trends with time (the differences in sampling would undermine such analysis). Descriptive analyses were done in Stata 14.2 (College Station, TX).

Since the main association analyzed - relating the mention of funding in press releases to news stories - is limited to the press releases with media coverage (n=429 for 2011, n=134 for 2015), we also give descriptive information for these subsets in Table 1. To examine the relationship between news and press releases, we used generalized estimating equations (GEE) to account for clustering of news stories for each press release (using an exchangeable working correlation; in SPSS, Version 23.). Odds ratios were converted to relative risks. For conflicts of interest, the association between press releases and news could not be analysed because so few news stories mentioned conflicts of interest.

Results

Disclosure of study funding and coi in the journal article

Among all 996 studies in 2011, 94% listed sources of funding in the journal article and 17% reported industry funding (**Table 1)**. The corresponding figures for 2015, among 254 studies, were 84% and 7%, respectively. In about one-quarter of studies (27% and 22% for 2011 and 2015), authors declared $\geq$ 1 conflict of interest (coi).

Disclosure of study funding and coi in the press release

Press releases reported a funding source 29% and 52% of the time (respectively for 2011 and 2015). Press releases specifically mentioned industry funding when promoting industry-funded studies 14% and 41% of the time, respectively. In the larger sample (2011

For peer review only - http://bmjopen.bmj.com/site/about/guidelines.xhtml

cohort) we could divide press releases issued by universities from those issued by journals; the universities were more likely to mention a funding source (59% vs 5%; absolute difference=54%, 95% CI: 49% to 59%). Reporting of conflicts of interest was rarer: 0% and 2% of press releases (for 2011 and 2015) mentioned a conflict of interest where one was declared in the journal article. Reporting of no conflicts was similarly rare: 0.4% and 0% of press releases explicitly reported no conflict for studies that explicitly declared none. For example, a university press release of an industry-funded study highlighted the authors' independence: "Painful periods increase sensitivity to pain throughout the month", included the statement "The study was funded by Pfizer and the Oxford Biomedical Research Centre. The researchers designed, carried out and reported the study independently of the funders".

Disclosure of study funding and coi in news stories

Reporting of funding sources in news stories was low: 9% for all studies; 17% for industry-funded studies in 2011; 10% for all studies and 0% for industry-funded studies in 2015. Reporting of conflicts of interest was even rarer: 1% and 0% of news stories (for 2011 and 2015) reported a conflict in the studies where a conflict was declared in the journal article, while 0.1% and 0% of news explicitly reported no conflict for studies that explicitly declared none.

Relationship of funding source in press release and the news

If the press release reported a funding source, associated news stories were more likely to report it (**Figure 1**). For the 2011 cohort, 22% of news stories reported funding if

For peer review only - http://bmjopen.bmj.com/site/about/guidelines.xhtml

in the press release vs 7% if not in the press release; Relative risk 3.1 (95% CI: 2.1 to 4.3); Absolute difference 15% (95% CI: 8% to 23%). For the 2015 cohort, 16% of news stories reported funding if in the press release vs 2% if not in the press release, RR 6.8 (95% CI: 2.2 to 17). The results were similar among the subset of 226 news from industry funded studies in 2011: 15% of news stories reported industry funding if in the press release vs 7% if not in the press release; Relative risk = 2.1 (95%CI 0.94 to 4.5); Absolute difference 8% (95%CI : 0% to 18%). For 2015, there were no reports in news of industry funding from the (much smaller) subset of industry funded studies (see Table 1).

Discussion

Our study highlights that reporting of funding sources is not high in either news or press releases from major biomedical journals and leading UK research universities. Neither was industry funding mentioned in the majority of news or press releases based on studies with industry funding. Mentioning conflicts of interest – or stating that there were none – was almost vanishingly rare. Note that we do not infer anything from the fluctuations between years. We were not attempting to analyse trends with time, and the two databases has some differences in sampling method that could confound such analysis. Rather the two cohorts simply illustrate the range of results from different samples.

We additionally observed that university press releases mentioned funding many more times than journal press releases did (in the cohort where we could analyse this). This difference deserves explanation, but we can only speculate. We believe authors and universities feel obliged (and are sometimes explicitly obliged) to acknowledge their funders – without whom the research could not have taken place. It is also likely that mentioning funders lends authority (to get funding, research projects must normally win a

For peer review only - http://bmjopen.bmj.com/site/about/guidelines.xhtml

highly selective competition). Journals have their own selective processes for publication, and appear not to feel the need to mention funders, either to acknowledge them or to enhance authority.

Consistent with prior work [3, 8-13], we observed that information - in this case funding source - is more likely to appear in the news when it is noted in the press release. Given that press releases are used as sources for news, this correlation is likely to contain a causal element, providing a potential means to increase the frequency with which news mentions funding and conflicts of interest, should authors and institutions wish to do so.

Disclosure of study funding and author conflicts interests matters: non-disclosure may undermine public and professional trust in the integrity of the research, while disclosure encourages readers to approach findings with appropriate skepticism. In the ‘post-truth’ era of mass information with varying credibility [16], it is particularly important for science and health research to be trusted.

Disclosure can only be effective if it reaches readers, most of whom - including many physicians [5] - learn about new research in the lay press. The level of underreporting that we observed may reflect the lack of explicit media policies about reporting industry funding [1]. We hope that this could change. It could be beneficial if press offices at medical journals, funders and academic institutions were to routinely highlight funding and disclosures in their press releases. One way to routinely operationalize this approach would be to add standard headers in press releases for funding and conflicts of interest as is done in many medical journals. Formal testing of alternate content and formats would inform the creation of more effective press releases. If press releases were made openly

For peer review only - http://bmjopen.bmj.com/site/about/guidelines.xhtml

available and linked to publications for peers to scrutinize, this might remind authors to declare their conflicts of interest.

A strength of this study is the large datasets of over 1200 press releases, 1200 journal articles and 1800 news when taken together. Several study limitations should be acknowledged. First, although we searched multiple databases attempting to target all major print and online news outlets, we may have missed some media coverage. While press releases for the leading UK academic universities and many leading Journals were covered, and there is no reason to suspect major differences between countries [9,10], or non-included journals, we cannot rule out that countries differ or that some journals or universities may have different press release policies. Further, since the association between press releases and news stories is observational, we suspect but cannot prove causation, nor can we be sure that this relationship is generalizable to all other media, such as social media. Another limitation is that statements about conflicts of interest in journal articles tend to focus on potential financial interests. Other potential conflicts can arise that are not stated [14,15], and thus not analysed here. For example, belonging to a professional organization or a research network or consortium can potentially result in entrenched viewpoints. Competition and reward structures within academia can also result in conflicts of interests.

In conclusion, we believe the research community's commitment to disclosing funding and conflicts of interest should extend to press releases - the most direct way that researchers communicate with the media. This does not seem to be the norm in most press releases issued by academic institutions and journals (at least in 2011 and 2015). It is likely

For peer review only - http://bmjopen.bmj.com/site/about/guidelines.xhtml

that including such information in press releases would raise the rate is it reported in news.

For peer review only

References

1. Hochman M, Hochman S, Bor D, et al. News media coverage of medication research: Reporting pharmaceutical company funding and use of generic medication names. *JAMA* 2008;300:1544-50.

2. Cook D, Boyd E, Grossmann C, et al. Reporting science and conflicts of interest in the lay press. *PLoS ONE* 2007 doi: 10.1371/journal.pone.0001266

3. Schwartz L, Woloshin S, Andrews A, et al. Influence of medical journal press releases on the quality of associated newspaper coverage: retrospective cohort study. *BMJ* 2011;344 doi: https://doi.org/10.1136/bmj.d8164

4. Schwitzer G. Trying to drink from a fire hose: Too much of the wrong kind of health care news. *Trends Pharmacol Sci* 2015;36:623-7.

5. Phillips D, Kanter E, Bednarczyk B, et al. Importance of the lay press in the transmission of medical knowledge to the scientific community. *N Engl J Med* 1991;325:1180-3.

6. Cary Funk, Brian Kennedy, and Meg Hefferon. Pew Research Center Report. Americans' views about public health studies in the news. February 2, 2017. Available at: http://www.pewinternet.org/2017/02/02/americans-views-about-public-health-and-health-studies-in-the-news/. Accessed September 25, 2017.

7. Yavchitz A, Boutron I, Bafeta A, et al.: Misrepresentation of randomized controlled trials in press releases and news coverage: a cohort study. *PLoS Med.* 2012;9(9):e1001308.

8. Sumner P, Vivian-Griffiths S, Boivin J, et al. The association between exaggeration in health related science news and academic press releases: retrospective observational study. *BMJ* 2014 doi: 10.1136/bmj.g7015

9. Sumner P, Vivian-Griffiths S, Boivin J, et al. Exaggerations and caveats in press release and health-related science news. *PLoS ONE* 2016 doi: https://doi.org/10.1371/journal.pone.0168217

For peer review only

10. Schat J, Bossema FG, Numans MN, SMeets, I, Burger, P. Overdreven gezondheidsnieuws. Relatie tussen overdrijving in academische persberichten en in nieuwsmedia. openaccess.leidenuniv.nl. 2018

11. Buhse S, Rahn AC, Bock M, et al.: Causal interpretation of correlational studies - Analysis of medical news on the website of the official journal for German physicians. *PLoS One.* 2018;13(5):e0196833. 29723258 10.1371/journal.pone.0196833 5933791

12. Adams RC, Challenger A, Bratton L, et al.: Claims of causality in health news: a randomised trial. *BMC Med.* 2019;17(1):91. 31092248 10.1186/s12916-019-1324-7 6521363

13. Bratton L, Adams RC, Challenger A, et al.: The association between exaggeration in health-related science news and academic press releases: a replication study. *Wellcome Open Research* 2019; 4:148 (https://doi.org/10.12688/wellcomeopenres.15486.2)

14. Birch K, Tyfield D. (2013) Theorizing the Bioeconomy: Biovalue, Biocapital, Bioeconomics or . . . What? Science Technol Human Values 38: 299-327.

15. Özdemir V, Kılıç H, Yıldırım A, et al. (2015) A code of ethics for ethicists: What would Pierre Bourdieu say? "Do not misuse social capital in the age of consortia ethics." American Journal of Bioethics 15: 64-67.

16. Lynch M. (2017) STS, symmetry and post-truth. Soc Stud Sci. 2017 Aug;47(4):593-599. doi: 10.1177/0306312717720308.

219

For peer review only

Table 1 Frequency of funding sources and conflicts of interest in journal articles, press releases and news reports

	All Studies		Studies with media coverage	
	**2011**	**2015**	**2011**	**2015**
**Information in journal article**	n=996	n=254	n=429	n=134
Funding source reported				
Any industry	17% (169/996)	7% (17/254)	19% (82/429)	7% (9/134)
Single non-industry sources				
Internal	4% (38/996)	8% (20/254)	4% (18/429)	8% (11/134)
Charity	13% (125/996)	6% (16/254)	13% (55/429)	8% (11/134)
Government	6% (56/996)	28% (71/254)	6% (24/429)	22% (30/134)
Multiple non-industry sources	55% (546/996)	41% (104/254)	52% (222/429)	42% (56/134)
None stated	6% (62/996)	16% (40/254)	7% (28/429)	18% (24/134)
Authors conflict of interest disclosed				
Declare "none"	57% (563/996)	50% (126/254)	54% (231/429)	48% (64/134)
Declare ≥ 1 conflict	27% (268/996)	22% (55/254)	29% (123/429)	21% (28/134)
No statement	16% (165/996)	29% (73/254)	17% (75/429)	31% (42/134)
**Information in press releases**				
Funding source reported				
Report any funding source				
All press releases	29% (284/996)	52% (131/254)	35% (150/429)	57% (76/134)
University	59% (253/426)	—	62% (127/206)	—
Journal	5% (31/570)	—	10% (23/223)	—
Report industry funding as % of studies with industry funding	14% (24/169)	41% (7/17)	24% (20/82)	44% (4/9)
Authors conflict of interest disclosed				
Report coi as % of studies where coi declared	0.5% (1/268)	2% (1/55)	0% (0/123)	4% (1/28)
Report no coi as % of studies that declared none	1% (4/563)	0% (0/126)	0.4% (1/231)	0% (0/64)
**Information in news stories**				
Funding source reported				
Report any funding	—	—	9% (112/1250)	10% (58/578)
Report industry funding as % of studies with industry funding	—	—	17% (38/226)	0% (0/27)
Authors conflict of interest disclosed				
Report coi as % of studies where coi declared	—	—	1% (4/380)	0% (0/114)
Report no coi as % of studies that declared none	—	—	0.1% (1/675)	0% (0/286)

For peer review only - http://bmjopen.bmj.com/site/about/guidelines.xhtml

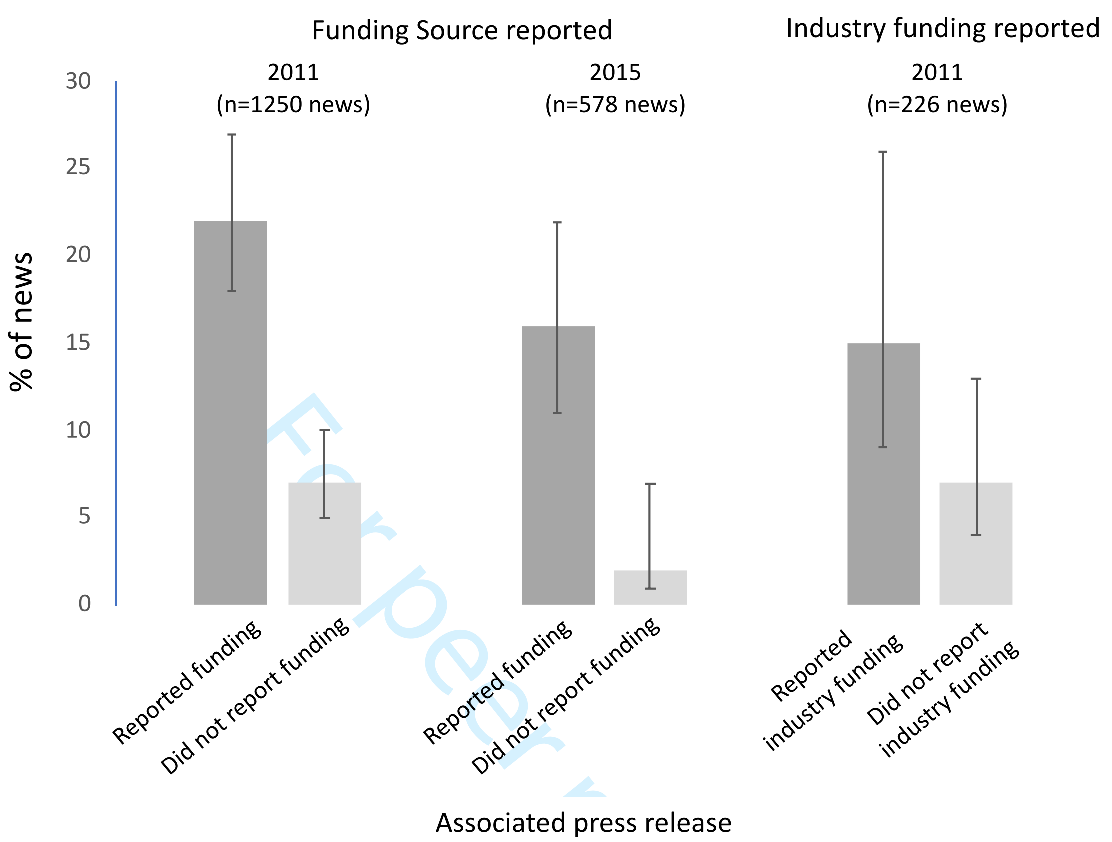

Figure 1. Proportion of news stories reporting study funding source or industry funding according to whether the associated press release reported the funding source or industry funding. There were zero mentions of industry funding in news in 2015 dataset, so analysis not performed. Error bars are 95% CI. The relative risks for the three plots are: 3.1 (95% CI: 2.1 to 4.3); 6.8 (95% CI: 2.2 to 17); 2.1 (95%CI 0.94 to 4.5).

For peer review only - http://bmjopen.bmj.com/site/about/guidelines.xhtml

BMJ Open

Disclosure of study funding and author conflicts of interest in press releases and the news: A retrospective content analysis with two cohorts.

Journal:	*BMJ Open*
Manuscript ID	bmjopen-2020-041385.R1
Article Type:	Original research
Date Submitted by the Author:	29-Oct-2020

Complete List of Authors:	Sumner, Petroc; Cardiff University School of Psychology,<br>Schwartz, Lisa; Dartmouth Institute for Health Policy and Clinical Practice<br>Woloshin, Steven; VA Medical Center / Dartmouth medical school, medicine<br>Bratton, Luke; Cardiff University School of Psychology<br>Chambers , Christopher; Cardiff University School of Psychology

**Primary Subject Heading**:	Public health
Secondary Subject Heading:	Communication
Keywords:	MEDICAL JOURNALISM, PUBLIC HEALTH, STATISTICS & RESEARCH METHODS

SCHOLARONE™
Manuscripts

I, the Submitting Author has the right to grant and does grant on behalf of all authors of the Work (as defined in the below author licence), an exclusive licence and/or a non-exclusive licence for contributions from authors who are: i) UK Crown employees; ii) where BMJ has agreed a CC-BY licence shall apply, and/or iii) in accordance with the terms applicable for US Federal Government officers or employees acting as part of their official duties; on a worldwide, perpetual, irrevocable, royalty-free basis to BMJ Publishing Group Ltd ("BMJ") its licensees and where the relevant Journal is co-owned by BMJ to the co-owners of the Journal, to publish the Work in this journal and any other BMJ products and to exploit all rights, as set out in our licence.

The Submitting Author accepts and understands that any supply made under these terms is made by BMJ to the Submitting Author unless you are acting as an employee on behalf of your employer or a postgraduate student of an affiliated institution which is paying any applicable article publishing charge ("APC") for Open Access articles. Where the Submitting Author wishes to make the Work available on an Open Access basis (and intends to pay the relevant APC), the terms of reuse of such Open Access shall be governed by a Creative Commons licence – details of these licences and which Creative Commons licence will apply to this Work are set out in our licence referred to above.

Other than as permitted in any relevant BMJ Author's Self Archiving Policies, I confirm this Work has not been accepted for publication elsewhere, is not being considered for publication elsewhere and does not duplicate material already published. I confirm all authors consent to publication of this Work and authorise the granting of this licence.

For peer review only

Disclosure of study funding and author conflicts of interest in press releases and the news: A retrospective content analysis with two cohorts.

Petroc Sumner, Professor, PhD[1]

Lisa M. Schwartz, MD, MS[2]

Steven Woloshin, MD, MS[2,3]

Luke Bratton, PhD[1]

Christopher D. Chambers, Professor, PhD[1]

[1]School of Psychology, Cardiff University, UK; sumnerp@cardiff.ac.uk

[2]The Dartmouth Institute for Health Policy and Clinical Practice, Lebanon, NH, USA

[3] The Lisa Schwartz Foundation for Truth in Medicine, Norwich, VT, USA

Corresponding author: Petroc Sumner, sumnerp@cardiff.ac.uk

For peer review only

Acknowledgements: This article is dedicated to the memory of Lisa Schwartz (1963-2018). Lisa inspired this investigation and produced the first version of the manuscript. Lisa died in November 2018. We believe this paper stays true to her spirit and intentions. We thank the following for data collection and related projects that informed our approach and interpretation here: Rachel Adams, Caitlin Argument, Amy Barrington, Jacky Boivin, Lewis Bott, Laura Benjamin, Hannah Coulson, Eleanor Corney, Bethan Dalton, Aimee Davies, Cecily Donnelly, Cameron Dunlop, Rebecca Emerson, Rose Fisher, Oliver Gray, Bethan Hughes, Katie John, Laura Jones, Sarah Mann, Olivia Manship, Hannah Maynard, Hannah McCarthy, Jack Ogden, Amy Parfitt, Naomi Scott, Lauren Stead, Christos Venetis, Solveiga Vivian-Griffiths, Eliza Walwyn-Jones, Claire Weeks, Leanne Whelan, Andrew Williams, Joe Wilton.

Funding - This project was supported by ESRC grant ES/M000664/1 and Wellcome grant 104943/Z/14/Z. The funders had no role in the analysis or writing of this paper.

Competing Interest Statement - The authors have no conflicts to declare.

Authorship PS, LMS, SW and CC designed the study based on LMS' suggestion. PS and CC collated the 2011 data and LB coded and collated the 2015 data. LMS and PS did the analyses. LMS wrote the first draft and PS wrote subsequent drafts with key contributions from SW, LB and CC.

Data sharing Data are publicly archived at https://osf.io/apc6d/

Word count: 2400

For peer review only - http://bmjopen.bmj.com/site/about/guidelines.xhtml

Objectives. To examine how often study funding and author conflicts of interest are stated in science and health press releases and in corresponding news; and whether disclosure in press releases is associated with disclosure in news. Second, to specifically examine disclosure rates in industry-funded studies.

Design. Retrospective content analysis with two cohorts.

Setting. Press releases about health, psychology or neuroscience research from research universities and journals from 2011 (n=996) and 2015 (n=254) and their associated news stories (n=1250 and 578).

Primary outcome measure. Mention of study funding and author conflicts of interest.

Results. In our 2011 cohort, funding was reported in 94% (934/996) of journal articles, 29% (284/996) of press releases and 9% (112/1250) of news. The corresponding figures for 2015 were: 84% (214/254), 52% (131/254) and 10% (58/578). A similar pattern was seen for the industry funding subset. If the press release reported study funding, news was more likely to: 22% if in the press release vs 7% if not in the press release (2011), RR 3.1 (95% CI: 2.1 to 4.3); for 2015, corresponding figures were 16% vs 2%, RR 6.8 (95% CI: 2.2 to 17). In journal articles, 27% and 22% reported a conflict of interest, while less than 2% of press releases or news ever mentioned these.

Conclusions. Press releases and associated news did not frequently state funding sources or conflicts of interest. Funding information in press releases was associated with such information in news. Given converging evidence that news draws on press release content, including statements of funding and conflicts of interest in press releases may lead to increased reporting in news.

For peer review only

Article Summary: Strengths and limitations of this study.

Strengths

- Reporting of study funding and conflicts of interest was assessed using a large cohort of press releases (1250) and news (1828) across two cohorts from separate years.
- The association between news and press release reporting was also assessed.

Limitations

- The study is correlational and retrospective
- The data included mainstream newspapers and internet media, but not broadcast media or social media.

For peer review only - http://bmjopen.bmj.com/site/about/guidelines.xhtml

- Generalisability to other countries and languages is unknown.

Introduction

Medical journals, funders, and academic institutions routinely call on researchers to disclose funding sources and financial conflicts of interest. Doing so is designed to increase the trustworthiness of the research process and allows readers to decide whether industry entanglements merit heightened skepticism when interpreting results.

There are no corresponding disclosure requirements for research reported in the lay press: only 3% of the largest circulation US newspapers had an explicit policy about reporting industry funding of medical research [1]. Published reports have documented substantial underreporting of author conflicts of interest and industry funding in the lay media [2-4]. Such underreporting matters since many people - including physicians [5] - learn about the results of medical research from the news [6].

The majority of news stories about news health-related discoveries are stimulated by press releases from universities or academic journals. Several studies suggest that press releases may strongly influence the content of subsequent media coverage. For example, news stories were more likely to report absolute risks, intervention harms and study limitations when they were reported in the medical journal press release [4]. Similarly, other aspects of news reports appear strongly associated with the wording and information in corresponding press releases, such as making causal claims from correlational data, exaggerating the relevance to humans of animal research, 'spin', or caveats to mitigate such exaggeration [7-13].

For peer review only - http://bmjopen.bmj.com/site/about/guidelines.xhtml

We analysed how often funding and conflicts of interest are mentioned in biomedical and health news stories and their corresponding journal and institution press releases. We examined whether the presence of such statements in press releases is associated with their presence in news. We then specifically examine the subset of studies that had industry funding.

Methods

Study design. We scrutinised two collections of health-related news stories, press releases and associated journal articles for reports of funding and conflicts of interest. We analysed the reporting frequencies and the association between reports in news and press releases.

Source materials. The first database contains 1250 news stories, 996 press releases and 996 associated journal articles [8,9]. This database was collated by selecting all the press releases related to human health published throughout 2011 from 8 leading international biomedical journals (Lancet, British Medical Journal (BMJ), Science, Nature, Nature Neuroscience, Nature Immunology, Nature Medicine and Nature Genetics) and 20 leading UK universities (The Russell Group; see **Figure 1**). The corresponding journal article for each press release was sourced, as were subsequent news stories in mainstream print and internet outlets [8,9]. The second database [12,13] contains 578 news stories, 254 press releases and 254 associated journal articles. This was collated by selecting press releases related to human health published between January and June 2015, from 26 UK universities (including the Russell Group and additional universities in Adams et al. 2019[12]) and 26 journals (10 journals affiliated with the BMJ group, 16 with the BMC group).

For peer review only

Search methods and inclusion criteria: Press releases were identified from publicly available repositories (web pages or EurekAlert) or nonpublic sites for journalists (Nature Publishing Group provided us with free access to all their press releases). The inclusion criteria were: health-related topic (broadly defined to include all biomedical sciences, diet, lifestyle, psychology) based on a peer-reviewed published journal article (that we could access). In the 2011 set all eligible press releases were included. In the 2015 set, the contribution from each institution had been capped to 10 press releases, selected randomly if more than 10 were available (for feasibility reasons [12,13]). If two press releases for the same journal article were identified (one from the university and one from the journal), only one was used, randomly selected. To identify any print and online news stories related to each press release, we searched Lexis-Nexis, BBC.co.uk, uk.reuters.com, and Google with keywords up to 28 days after publication of the press release, and up to one week before (to allow for potential news before the embargo was lifted).

Data extraction and coding. Journal articles, press releases and related news coverage were coded by research assistants using a pre-specified protocol, to extract information about study funding and the authors' reported conflicts of interest. Study funding was coded as *industry* (e.g. GlaxoSmithKline, Pfizer etc.), *government* (e.g. the research councils such as the NIH and US National Cancer Institute, MRC, BBSRC, ESRC etc.), *charity* (e.g. Wellcome Trust, Cancer Research UK, the British Heart Foundation etc.), *internal/other* (e.g. self- or university-funded), or *none mentioned*. The first-mentioned source was always coded, and then industry funding if it was listed. Therefore studies could belong to more

For peer review only

than one category (e.g. *government* and *industry*). For the specific analysis of industry funding, a study was included if an industry source was mentioned, regardless of position in the list of funders. Coders located the "Conflict of interest" or "Competing interests" sections of the article and determined whether there was no declaration found, a declaration of no conflict of interest (e.g. "The authors declare no conflict of interest") or whether any author declared conflicts of interest (e.g. "Author X is a paid consultant to Y company).

The press releases and news stories were simply coded for whether funding or conflicts of interest were reported, and whether the press release and news specifically mentioned any industry funding.

The raw materials and protocols for the two databases are available at https://figshare.com/articles/dataset/InSciOut/903704 and https://osf.io/apc6d/. The latter also contains the extracted data used for this study, in the folder 'Processed data / funding and conflicts of interest'.

Coding reliability. For the 2011 set, a second research assistant independently coded a randomly selected sample of 28% of press releases and associated news (23% of total news stories). Observed agreement was 94% for coding the type of funding source, 92% for whether PR reported funding, 94% for whether news reported funding, 98% for the study's conflict of interest statement, and 99% for both whether press release and news mentioned conflict of interest (we do not calculate kappa as it is unreliable when agreement is this high). For coding disagreements, one answer was randomly selected. For the 2015 set, a second research assistant independently coded all texts, and any discrepancies were

For peer review only

subsequently highlighted and discussed to reach a consensus conclusion. A third research assistant arbitrated if disagreements remained (very rare).

Analysis. We first report analyses of all journal articles and associated press releases descriptively, followed by descriptive analysis of news. These are separated by year to illustrate the natural range of fluctuation, rather than to examine trends with time (the differences in sampling would undermine such analysis). Descriptive analyses were done in Stata 14.2 (College Station, TX). Since the main association analyzed - relating the mention of funding in press releases to news stories - is limited to the press releases with media coverage, we also give descriptive information for these subsets in Table 1. To examine the relationship between news and press releases, we used generalized estimating equations (GEE) to account for clustering of news stories for each press release (using an exchangeable working correlation; in SPSS, Version 23.)s. For conflicts of interest, the association between press releases and news could not be analysed because so few news stories mentioned conflicts of interest.

Patient and public involvement. No patients or participants were involved in this study.

Results

Disclosure of study funding and conflicts of interest in the journal article

Among all 996 studies in 2011, 94% (934) listed sources of funding in the journal article and 17% (169) reported industry funding . The corresponding figures for 2015, among 254 studies, were 84% (214) and 7% (17), respectively. In about one-quarter of studies (27%

For peer review only - http://bmjopen.bmj.com/site/about/guidelines.xhtml

and 22% for 2011 and 2015), one or more authors declared a conflict of interest (see Table 1 for all numbers and %).

Disclosure of study funding and conflicts of interest in the press release

Press releases reported a funding source 29% and 52% of the time (respectively for 2011 and 2015). Press releases specifically mentioned industry funding when promoting industry-funded studies 14% and 41% of the time, respectively. In the larger sample (2011 cohort) we could divide press releases issued by universities from those issued by journals; the universities were more likely to mention a funding source (59% vs 5%; absolute difference=54%, 95% CI: 49% to 59%). Reporting of conflicts of interest was rarer: 0% and 2% of press releases (for 2011 and 2015) mentioned a conflict of interest where one was declared in the journal article. Reporting of no conflicts was similarly rare: 0.4% and 0% of press releases explicitly reported no conflict for studies that explicitly declared none.

Disclosure of study funding and conflicts of interest in news stories

For the set of studies with media coverage, reporting of funding sources in news stories was low: 9% for all studies; 17% for industry-funded studies in 2011; 10% for all studies and 0% for industry-funded studies in 2015. Reporting of conflicts of interest was even rarer: 1% and 0% of news stories (for 2011 and 2015) reported a conflict in the studies where a conflict was declared in the journal article, while 0.1% and 0% of news explicitly reported no conflict for studies that explicitly declared none.

Relationship of funding source in press release and the news

For peer review only - http://bmjopen.bmj.com/site/about/guidelines.xhtml

If the press release reported a funding source, associated news stories were more likely to report it (**Figure 2**). For the 2011 cohort, 22% of news stories reported funding if in the press release vs 7% if not in the press release; Relative risk 3.1 (95% CI: 2.1 to 4.3); Absolute difference 15% (95% CI: 8% to 23%). For the 2015 cohort, 16% of news stories reported funding if in the press release vs 2% if not in the press release, RR 6.8 (95% CI: 2.2 to 17). The results were similar among the subset of 226 news from industry funded studies in 2011: 15% of news stories reported industry funding if in the press release vs 7% if not in the press release; Relative risk = 2.1 (95%CI 0.94 to 4.5); Absolute difference 8% (95%CI : 0% to 18%). For 2015, there were no reports in news of industry funding from the subset of industry funded studies (n= 9 studies, 27 news; see Table 1).

Discussion

Our study highlights that reporting of funding sources is not high in either news or press releases from major biomedical journals and leading UK research universities. Neither was industry funding mentioned in the majority of news or press releases based on studies with industry funding. Mentioning conflicts of interest – or stating that there were none – was almost vanishingly rare.

Consistent with prior work [4, 8-13], we observed that information - in this case funding source - is more likely to appear in the news when it is noted in the press release. Given that press releases are used as sources for news, we believe this correlation is likely to contain a causal element. In turn, this would provide a means to increase the frequency with which news mentions funding and conflicts of interest, should authors and institutions wish to do so (or develop a policy to do so).

For peer review only - http://bmjopen.bmj.com/site/about/guidelines.xhtml

Disclosure of study funding and author conflicts interests matters: non-disclosure may undermine public and professional trust in the integrity of the research, while disclosure is designed to allow readers to approach findings with appropriate skepticism. In an era of mass information with varying credibility, it is particularly important for science and health research to be trustworthy.

Disclosure can only be effective if it reaches readers, most of whom - including many physicians [5] - learn about new research in the lay press. The level of underreporting that we observed may reflect the lack of explicit media policies about reporting industry funding [1]. We hope that this could change. It could be beneficial if press offices at medical journals, funders and academic institutions were to routinely highlight funding and disclosures in their press releases. One way to routinely operationalize this approach would be to add standard headers in press releases for funding and conflicts of interest as is done in many medical journals. Formal testing of alternate content and formats would inform the creation of more effective press releases. If press releases were made openly available and linked to publications for peers to scrutinize, this might remind authors to declare their conflicts of interest.

A strength of this study is the large datasets of over 1200 press releases, 1200 journal articles and 1800 news when taken together. Several study limitations should be acknowledged. First, since the association between press releases and news stories is observational, we cannot prove causation. Second, we do not infer anything from the fluctuations between years. We were not attempting to analyse trends with time, because the two databases have some differences in sampling method that could confound such analysis. Rather the two cohorts simply illustrate the range of results from different

For peer review only - http://bmjopen.bmj.com/site/about/guidelines.xhtml

samples. Third, although we searched multiple databases attempting to target all major print and online news outlets, we did not include broadcast media, and we may have missed some media coverage. Fourth , the extent of generalisability is uncertain; while press releases for the leading UK academic universities and many leading Journals were covered, and there is no reason to suspect major differences between countries [9,10], or non-included journals, we cannot rule out that countries differ or that some journals or universities may have different press release policies, nor can we be sure that this relationship is generalizable to all other media, such as social media. Fifth, statements about conflicts of interest in journal articles tend to focus on potential financial interests; non-financial interests can arise that are not stated [14-16], and thus not analysed here. For example, belonging to a professional organization or a research network or consortium can potentially result in entrenched viewpoints, while competition and reward structures within academia can also result in conflicts of interests. Lastly, we simplified our coding to whether funding or conflicts were present or absent in press releases and news, and did not capture whether reporting fairly represented the entire set of funding or COI in the study.

We additionally observed that university press releases mentioned funding many more times than journal press releases did (in the 2011 cohort where we could analyse this, see Table 1). This difference deserves explanation, but we can only speculate. We believe authors and universities feel obliged (and are sometimes explicitly obliged) to acknowledge their funders – without whom the research could not have taken place. It is also likely that mentioning funders lends authority (to get funding, research projects must

For peer review only - http://bmjopen.bmj.com/site/about/guidelines.xhtml

normally win a highly selective competition). Journals have their own selective processes for publication, and appear not to feel the need to mention funders, either to acknowledge them or to enhance authority. We hope that journals will adopt policies to highlight funding and conflicts of interest in their press releases.

In conclusion, we believe the research community's commitment to disclosing funding and conflicts of interest should extend to press releases - the most direct way that researchers communicate with the media. This does not seem to be the norm in most press releases issued by academic institutions and journals (at least in 2011 and 2015). It is likely that including such information in press releases would raise the rate is it reported in news.

For peer review only

References

1. Hochman M, Hochman S, Bor D, et al. News media coverage of medication research: Reporting pharmaceutical company funding and use of generic medication names. *JAMA* 2008;300:1544-50.

2. Moynihan R, et. al. Coverage by the news media of the benefits and risks of medications, N Engl J Med 2000; 342:1645-1650

3. Cook D, Boyd E, Grossmann C, et al. Reporting science and conflicts of interest in the lay press. *PLoS ONE* 2007 doi: 10.1371/journal.pone.0001266

4. Schwartz L, Woloshin S, Andrews A, et al. Influence of medical journal press releases on the quality of associated newspaper coverage: retrospective cohort study. *BMJ* 2011;344 doi: https://doi.org/10.1136/bmj.d8164

5. Phillips D, Kanter E, Bednarczyk B, et al. Importance of the lay press in the transmission of medical knowledge to the scientific community. *N Engl J Med* 1991;325:1180-3.

6. Cary Funk, Brian Kennedy, and Meg Hefferon. Pew Research Center Report. Americans' views about public health studies in the news. February 2, 2017. Available at: http://www.pewinternet.org/2017/02/02/americans-views-about-public-health-and-health-studies-in-the-news/. Accessed September 25, 2017.

7. Yavchitz A, Boutron I, Bafeta A, et al.: Misrepresentation of randomized controlled trials in press releases and news coverage: a cohort study. *PLoS Med.* 2012;9(9):e1001308.

8. Sumner P, Vivian-Griffiths S, Boivin J, et al. The association between exaggeration in health related science news and academic press releases: retrospective observational study. *BMJ* 2014 doi: 10.1136/bmj.g7015

9. Sumner P, Vivian-Griffiths S, Boivin J, et al. Exaggerations and caveats in press release and health-related science news. *PLoS ONE* 2016 doi: https://doi.org/10.1371/journal.pone.0168217

10. Schat J, Bossema FG, Numans MN, SMeets, I, Burger, P. Overdreven gezondheidsnieuws. Relatie tussen overdrijving in academische persberichten en in nieuwsmedia. openaccess.leidenuniv.nl. 2018

For peer review only - http://bmjopen.bmj.com/site/about/guidelines.xhtml

11. Buhse S, Rahn AC, Bock M, et al.: Causal interpretation of correlational studies - Analysis of medical news on the website of the official journal for German physicians. *PLoS One.* 2018;13(5):e0196833. 29723258 10.1371/journal.pone.0196833 5933791

12. Adams RC, Challenger A, Bratton L, et al.: Claims of causality in health news: a randomised trial. *BMC Med.* 2019;17(1):91. 31092248 10.1186/s12916-019-1324-7 6521363

13. Bratton L, Adams RC, Challenger A, et al.: The association between exaggeration in health-related science news and academic press releases: a replication study. *Wellcome Open Research* 2019; 4:148 (https://doi.org/10.12688/wellcomeopenres.15486.2)

14. Birch K, Tyfield D. (2013) Theorizing the Bioeconomy: Biovalue, Biocapital, Bioeconomics or . . . What? Science Technol Human Values 38: 299-327.

15. Özdemir V, Kılıç H, Yıldırım A, et al. (2015) A code of ethics for ethicists: What would Pierre Bourdieu say? "Do not misuse social capital in the age of consortia ethics." American Journal of Bioethics 15: 64-67.

16. Bero, L.A., Grundy, Q., 2016. Why Having a (Nonfinancial) Interest Is Not a Conflict of Interest. PLOS Biology 14, e2001221. doi:10.1371/journal.pbio.2001221

232

For peer review only - http://bmjopen.bmj.com/site/about/guidelines.xhtml

Table 1 Frequency of funding sources and conflicts of interest in journal articles, press releases and news reports. Note that studies could belong to more than one funding category (e.g. *government* and *industry*).

	All Studies		Studies with media coverage	
	**2011**	**2015**	**2011**	**2015**
**Information in journal article**	N=996	N=254	N=429	N=134
Funding source reported				
Any funding reported	94% (934/996)	84% (214/254)	93% (401/429)	82% (110/134)
Any industry	17% (169/996)	7% (17/254)	19% (82/429)	7% (9/134)
Single non-industry sources				
Government	6% (56/996)	28% (71/254)	6% (24/429)	22% (30/134)
Charity	13% (125/996)	6% (16/254)	13% (55/429)	8% (11/134)
Internal/other	4% (38/996)	8% (20/254)	4% (18/429)	8% (11/134)
Multiple non-industry sources	55% (546/996)	41% (104/254)	52% (222/429)	42% (56/134)
None stated	6% (62/996)	16% (40/254)	7% (28/429)	18% (24/134)
Authors conflict of interest disclosed				
Declare "none"	57% (563/996)	50% (126/254)	54% (231/429)	48% (64/134)
Declare ≥ 1 conflict	27% (268/996)	22% (55/254)	29% (123/429)	21% (28/134)
No statement	16% (165/996)	29% (73/254)	17% (75/429)	31% (42/134)
**Information in press releases**				
Funding source reported				
Report any funding source				
All press releases	29% (284/996)	52% (131/254)	35% (150/429)	57% (76/134)
University	59% (253/426)	—	62% (127/206)	—
Journal	5% (31/570)	—	10% (23/223)	—
Report industry funding as % of studies with industry funding	14% (24/169)	41% (7/17)	24% (20/82)	44% (4/9)
Authors conflict of interest disclosed				
Report coi as % of studies where coi declared	0.5% (1/268)	2% (1/55)	0% (0/123)	4% (1/28)
Report no coi as % of studies that declared none	1% (4/563)	0% (0/126)	0.4% (1/231)	0% (0/64)
**Information in news stories**				
Funding source reported				
Report any funding	—	—	9% (112/1250)	10% (58/578)
Report industry funding as % of studies with industry funding	—	—	17% (38/226)	0% (0/27)
Authors conflict of interest disclosed				
Report coi as % of studies where coi declared	—	—	1% (4/380)	0% (0/114)

For peer review only - http://bmjopen.bmj.com/site/about/guidelines.xhtml

Report no coi as % of studies that declared none	—	—	0.1% (1/675)	0% (0/286)

Figure 1. Flow diagram describing the two datasets and the available numbers for analysis.

Figure 2. Proportion of news stories reporting study funding source or industry funding according to whether the associated press release reported the funding source or industry funding. There were zero mentions of industry funding in news in 2015 dataset, so analysis not performed. Error bars are 95% CI. The relative risks for the three plots are: 3.1 (95% CI: 2.1 to 4.3); 6.8 (95% CI: 2.2 to 17); 2.1 (95%CI 0.94 to 4.5).

For peer review only

For peer review only - http://bmjopen.bmj.com/site/about/guidelines.xhtml

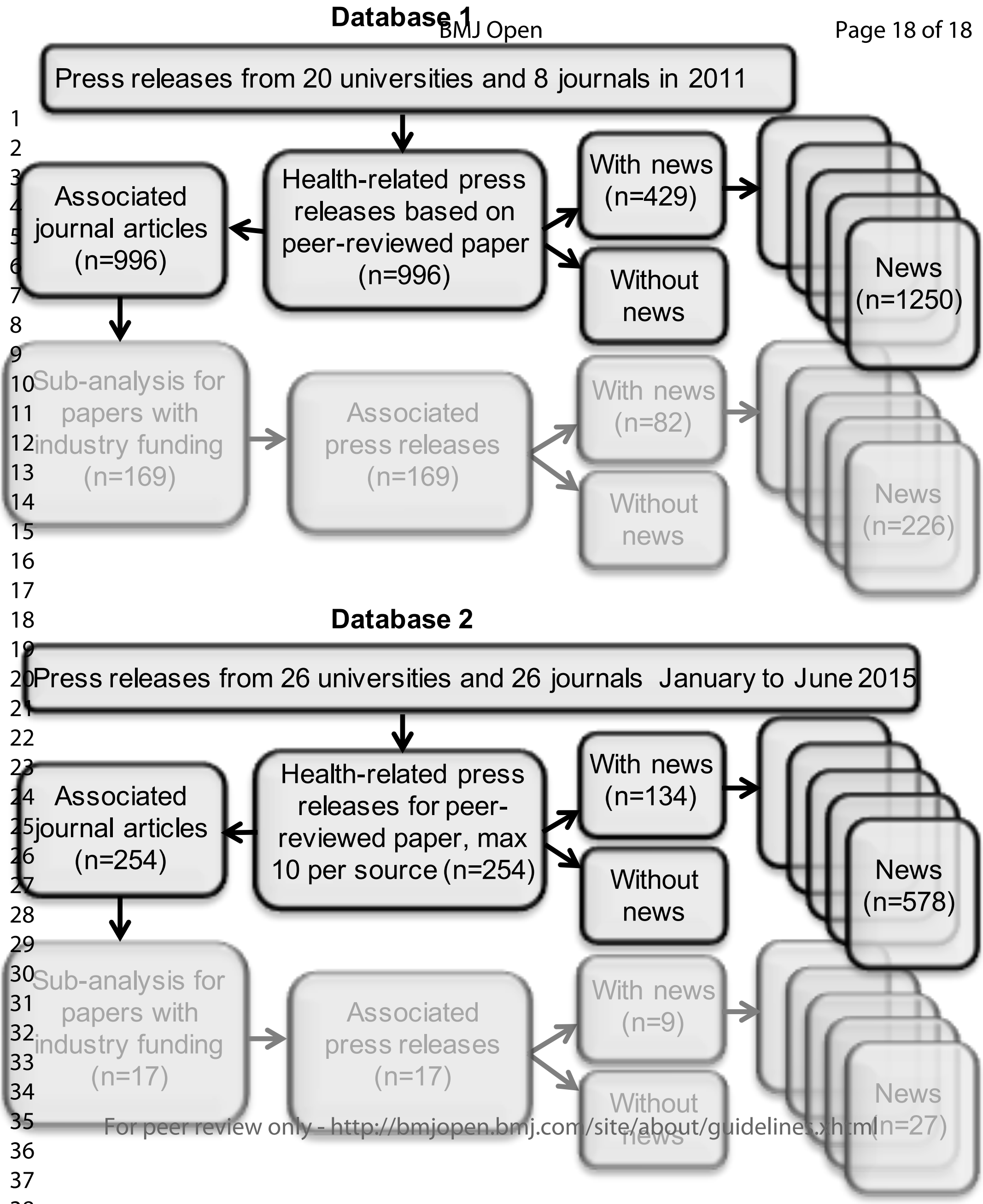
Database 1
Press releases from 20 universities and 8 journals in 2011
Associated journal articles (n=996)
Health-related press releases based on peer-reviewed paper (n=996)
With news (n=429)
Without news
News (n=1250)
Sub-analysis for papers with industry funding (n=169)
Associated press releases (n=169)
With news (n=82)
Without news
News (n=226)
Database 2
Press releases from 26 universities and 26 journals January to June 2015
Associated journal articles (n=254)
Health-related press releases for peer-reviewed paper, max 10 per source (n=254)
With news (n=134)
Without news
News (n=578)
Sub-analysis for papers with industry funding (n=17)
Associated press releases (n=17)
With news (n=9)
Without news
News (n=27)

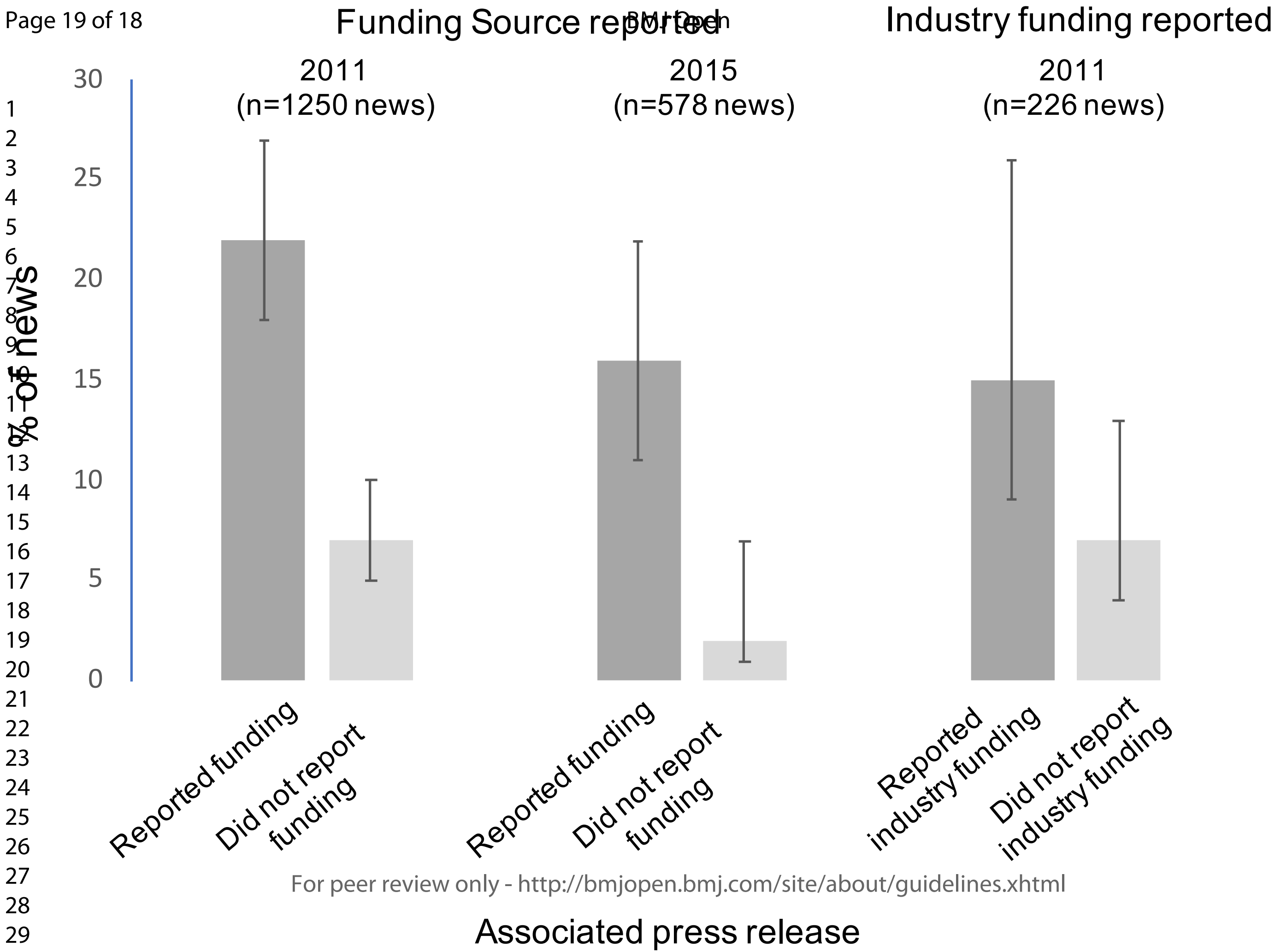

For peer review only - http://bmjopen.bmj.com/site/about/guidelines.xhtml
